# Water Relations, Gas Exchange, Chlorophyll Fluorescence and Electrolyte Leakage of Ectomycorrhizal *Pinus halepensis* Seedlings in Response to Multi-Heavy Metal Stresses (Pb, Zn, Cd)

**DOI:** 10.3390/microorganisms10010057

**Published:** 2021-12-28

**Authors:** Chadlia Hachani, Mohammed S. Lamhamedi, Abdenbi Zine El Abidine, Mejda Abassi, Damase P. Khasa, Zoubeir Béjaoui

**Affiliations:** 1 Faculty of Sciences of Bizerte, University of Carthage, Jarzouna 7021, Tunisia; chadliahachanii@gmail.com; 2 Laboratory of Forest Ecology (LR11INRGREF03), National Institute of Research in Rural Engineering, Water and Forests (INRGREF), University of Carthage, Hédi Elkarray Street, Elmenzah IV, BP 10, Ariana 2080, Tunisia; mej_abassi@yahoo.fr; 3 Centre for Forest Studies, Faculty of Forestry, Geography and Geomatics, Abitibi Price Building, Laval University, Quebec, QC G1V 0A6, Canada; mohammed-sghir.lamhamedi.1@ulaval.ca; 4 National Forest School of Engineers, B.P. 5 1 1, Tabriquet, Salé 11015, Morocco; zineenfi@gmail.com; 5 Centre for Forest Research and Institute for Systems and Integrative Biology, Université Laval, 1030 Avenue de la Médecine, Quebec, QC G1V 0A6, Canada; damase.khasa@ibis.ulaval.ca

**Keywords:** *Pinus halepensis*, *Rhizopogon*, metal stress, osmotic adjustment, net photosynthesis, tolerance

## Abstract

The success of mine site restoration programs in arid and semi-arid areas poses a significant challenge and requires the use of high-quality seedlings capable of tolerating heavy metal stresses. The effect of ectomycorrhizal fungi on different physiological traits was investigated in *Pinus halepensis* seedlings grown in soil contaminated with heavy metals (Pb-Zn-Cd). Ectomycorrhizal (M) and non-ectomycorrhizal (NM) seedlings were subjected to heavy metals stress (C: contaminated, NC: control or non-contaminated) soils conditions for 12 months. Gas exchange, chlorophyll fluorescence, water relations parameters derived from pressure–volume curves and electrolyte leakage were evaluated at 4, 8 and 12 months. Ectomycorrhizal symbiosis promoted stronger resistance to heavy metals and improved gas exchange parameters and water-use efficiency compared to the non-ectomycorrhizal seedlings. The decrease in leaf osmotic potentials (Ψ_π_^100^: osmotic potential at saturation and Ψ_π_^0^: osmotic potential with loss of turgor) was higher for M-C seedling than NM-C ones, indicating that the ectomycorrhizal symbiosis promotes cellular osmotic adjustment and protects leaf membrane cell against leakage induced by Pb, Zn and Cd. Our results suggest that the use of ectomycorrhizal symbiosis is among the promising practices to improve the morphophysiological quality of seedlings produced in forest nurseries, their performance and their tolerance to multi-heavy metal stresses.

## 1. Introduction

Mining activities that are conducted in a Mediterranean climate can exert their greatest effects on the environment through water pollution, contamination and alteration of agricultural soil due to the spread of heavy metals [1,2]. In North Africa, land losses due to increasingly heavy metal-polluted soils are the highest in the world [1]. Therefore, rehabilitation strategies targeting mine-degraded areas have been launched to combat land degradation and have been recognized as one of several sustainable development goals [1]. However, phytoremediation of abandoned mine lands by installing forest plantation presents a great challenge [3]. Indeed, severe environmental stresses and their interactions can negatively affect the survival of forest trees [4,5,6], the sustainability of ecosystems and the success of reforestation programs on abandoned mine sites in the context of climate change. North Africa (Morocco, Algeria and Tunisia) is recognized as one of the Mediterranean region’s most vulnerable to climate change, given that it is characterized by a significant decrease in precipitation and a significant increase in temperature, which reinforce both the pressures and the phenomena that are associated with ecosystem degradation [5,7].

In North Africa, various efforts have been made to modernize the seedling production chain and forest nurseries that produce seedlings of high morphophysiological quality, which are capable of surviving, growing and tolerating various environmental stresses in reforestation sites [8,9,10]. The results of this modernization project have shown that growth of ectomycorrhizal seedlings, which are produced in containers in modern forest nurseries, was much higher than that of seedlings that were produced in polybags in traditional nurseries [9]. Yet, these restoration projects did not focus upon the rehabilitation of mining sites using adapted species and ectomycorrhizal seedlings that were produced in modern forest nurseries.

The development of mycorrhizoremediation technology involving the use ectomycorrhizal seedlings is seen as a way for enhancing their abilities to tolerate various multi-metal stresses [11,12,13]. Other studies have reported the efficiency of ectomycorrhizal seedlings in overcoming the detrimental effects of abiotic stresses, such as drought [14,15] and salinity [16,17]. In contrast, numerous studies have demonstrated the negative effects of heavy metal contamination on the survival and growth of a wide range of non-mycorrhizal plant species [18,19,20,21,22,23]. On one hand, an excess of heavy metals in soils limits the efficiency of water- and mineral nutrient-use [18,19,24]. In addition, heavy metals substantially reduce leaf gas exchange parameters (net photosynthesis, transpiration, stomatal conductance, etc.), thereby causing lower water flow from the soil to the leaves [18,25], which may cause water stress in seedlings [24]. On the other hand, CO_2_ assimilation and leaf transpiration are decreased due to reduced stomatal opening [26,27], which may be induced by direct interaction of metal ion toxicity with guard cells [19]. Furthermore, long-term exposure to high levels of toxic heavy metals is often followed by water deficits [19,28]. This leads to the appearance of physiological responses consistent with those found under drought stress, including reductions in root water uptake, leaf turgor and stomatal conductance [19,29,30]. Heavy metals, such as Pb, Zn and Cd, reduce cell membrane permeability [18,31], chlorophyll concentrations [32,33] and photosystem II activity [25], which in turn can limit photosynthesis leading to metabolic disruptions [27,34,35].

To improve the survival, growth and physiological processes of tree seedlings that are intended for reforestation and mine site restoration programs, the use of seedlings with high morphophysiological quality that are produced in modern forest nurseries is necessary [8,9,36]. In addition to the choice of local forest species that are already adapted to the interactions of different environmental stresses, improving the root system using compatible host-ectomycorrhizal fungi that are resistant to environmental stresses would improve the survival, growth and physiology of the seedlings after their installation in mining and reforestation sites. Under drought conditions, as is the case in arid and semi-arid areas of North Africa, it was shown that ectomycorrhizal seedlings with fungal genotypes that produce mycelial strands or rhizomorphs capable of transporting large amounts of water [37] can substantially improve physiological processes (gas exchange and water relations parameters, mineral nutrition and hydraulic conductivity of the roots, among others). Consequently, the drought tolerance of the seedlings is improved [9,14,38,39,40]. In contrast, it is recognized that in the presence of heavy metals, ectomycorrhizal fungi improve survival, growth and various physiological processes (transpiration, gas exchange parameters: stomatal conductance, transpiration, net photosynthesis and osmotic adjustment, among others) of forest seedlings under controlled conditions and on mining sites [11,41,42]. Despite the publication of several reviews on the water relations of different species in response to heavy metal stresses [18,19,24,43], no information has been made available, to our knowledge, regarding ectomycorrhizal effects on water relations parameters that are derived from pressure–volume curves [44,45,46] in response to heavy metal stresses (Pb, Zn and Cd). These water relations variables for ectomycorrhizal tree seedlings, include osmotic potential at saturation (Ψ_π_^100^), osmotic potential with loss of turgor (Ψ_π_^0^), relative water content at loss of turgor (RWC_0_), water content of the symplasm (SWC), the modulus of elasticity (ε_max_) and osmotic adjustment (OA). The determination of these variables that are specific to water relations is fundamental to better quantifying and understanding the effects of ectomycorrhizal fungi on the physiology of cells and tissues of forest seedlings, which allow them to survive, grow and maintain their physiological functions (net photosynthesis, transpiration, etc.) in response to multi-heavy metal stresses. In addition, the results of this study would help advance operational practices, thereby further improving the morphophysiological quality of seedlings that are produced in forest nurseries, together with their performance in mining sites that are located in arid and semi-arid regions. This research continues our recent work [47], which showed that ectomycorrhizal fungi (*Rhizopogon* sp.) improved growth and mineral nutrient contents of *Pinus halepensis* mill seedlings that were subjected to heavy metal stresses. The presence of ectomycorrhizal fungi also reduced translocation factors for Zn and Cd, and bioaccumulation factors for Pb and Cd. This study was designed to test the hypothesis that ectomycorrhizal fungi can improve the water relations and the gas exchange parameters of *P. halepensis* seedlings grown in response to multi-heavy metal stresses.

The objectives of this study were: (i) to compare gas exchange variables, water use-efficiency (WUE), electrolyte leakage and chlorophyll fluorescence under multiple heavy metal stresses (Pb, Zn and Cd) in ectomycorrhizal and non-ectomycorrhizal *Pinus halepensis* seedlings; (ii) to determine water relations parameters that were derived from pressure–volume curves (Ψ_π_^100^, Ψ_π_^0^, RWC_0_, SWC and ε_max_) of ectomycorrhizal and non-ectomycorrhizal seedlings that were grown in the absence and presence of multi-metal stresses; and (iii) to examine whether osmotic adjustment occurs as a result of long-term exposure of ectomycorrhizal seedlings to high levels of heavy metal toxicity. This evaluation of gas exchange and water relations parameters will help to understand the physiological processes relevant to the performance of plants in response to multi-heavy metal stresses.

## 2. Materials and Methods

### 2.1. Soils, Plant Material, Experimental Design and Growth Conditions

Contaminated soil samples were collected from the abandoned mine site of “Jebel Ressas” in North Tunisia (36°36′021.4″ N, 10°19′04.0″ E) to a depth of 20 cm, as described by Hachani et al. [47]. Control soil was collected from a non-contaminated area, as described by Hachani et al. [47]. Heavy metal concentrations were determined by Inductively Coupled Plasma-Optical Emission Spectroscopy (ICP-OES) using three soil composite samples (six soil samples per composite sample) [47]. Heavy metal concentrations were 15.587 ± 0.796 mg·g^−1^ (mean ± standard deviation) for Pb, 37.766 ± 3.210 mg·g^−1^ for Zn and 0.181 ± 0.033 mg.g^−1^ for Cd in contaminated soil. In control soil, metal concentrations were 0.009 ± 0.0004 mg·g^−1^ and 0.021 ± 0.002 mg·g^−1^ for Pb and Zn, respectively, while Cd concentrations were at or below detection limits [47]. The other physicochemical characteristics of the contaminated and control soils (pH_water_, pH_CaCl2_, electrical conductivity, soil fertility, etc.) that were used in this study are described in detail in our previous study [47].

The experiment was conducted at the National Institute of Research in Rural Engineering, Water and Forests (INRGREF) in Tunis (Tunisia). Nine month old naturally ectomycorrhizal and non-ectomycorrhizal Aleppo pine (*Pinus halepensis*) seedlings were sampled from a modern forest nursery (Ouchtata) in northwestern Tunisia (36°57′40.3″ N, 8°59′50.0″ E). The seedlings were divided into two classes according to the degree of the surface colonization of their root plugs by the extraradical mycelium of the ectomycorrhizal fungus *Rhizopogon* sp., as described in our previous studies [9,47,48]. The first class included seedlings with no surface colonization (non-mycorrhizal seedlings), while the second included seedlings with more than 50% of their root plug areas that were covered by the extramatrical phase of the fungus. The initial growth variables of the different parts of the *Pinus halepensis* seedlings that were used in this study are described in detail by Hachani et al. [47].

The selected seedlings were transplanted into pots (volume: 2 L; height: 45 cm; diameter: 30 cm) that were filled with contaminated (C) and non-contaminated (NC) soil at the rate of 10 kg of soil/pot (one seedling/pot). The pots containing the seedlings of the four treatments were grown for 12 months and they were installed according to a 2 × 2 factorial experimental design in four complete random blocks. These four treatments include: NM-NC (non-mycorrhizal seedlings + non-contaminated soil); M-NC (mycorrhizal seedlings + non-contaminated soil); NM-C (non-mycorrhizal seedlings + contaminated soil); and M-C (mycorrhizal seedlings + contaminated soil). The treatments were randomly distributed in each block. A total of 320 seedlings were deployed at the rate of 20 seedlings/treatment/block (20 seedlings × 4 treatments × 4 blocks). For each pot, soil moisture was maintained at 78 ± 6% field capacity throughout the experiment using Time Domain Reflectometry (TDR, Trase system I, Soil Moisture Equipment Corp., Goleta, CA, USA) [49,50].

### 2.2. Gas Exchange, Water-Use Efficiency and Chlorophyll Fluorescence Measurements

Maximal gas exchange variables were measured on five randomly selected seedlings/treatment/block. Measurements were carried out on three dates (4, 8 and 12 months), between 09.30 and 11.30 solar time to ensure maximum photosynthetic assimilation [51]. Net photosynthesis (A), stomatal conductance (g_s_), transpiration (E) and intercellular CO_2_ concentration (C_i_) were measured using an infrared analyzer (LCpro+, ADC Bio Scientifc Ltd., Hoddesdon, UK) with a cylindrical coniferous cuvette at an active photosynthetic photon flux density (PPFD) of 984 ± 56 µmol m^−2^ s^−1^, a CO_2_ concentration of 350 µmol mol^−1^ and a leaf temperature of 29 ± 4 °C. Water-use efficiency (WUE) was calculated as the ratio of net photosynthesis to transpiration [52]. The gas exchange variables were corrected by taking into account the leaf area of the needles, which were estimated using the approach described by Lamhamedi et al. [49].

Chlorophyll fluorescence was determined on the same seedlings used for gas exchange measurements using a PEA fluorometer (Plant Efficiency Analyzer, Hansatech, King’s Lynn, Norfolk, UK). Measurements of minimum (F_0_) and maximum (F_m_) chlorophyll fluorescence were performed on attached needles after 1 h of dark adaptation. The potential quantum yield of photosystem II (PSII), which was expressed as F_v_/F_m_, was calculated as follows [53]:

F_v_/F_m_ = (F_m_ − F_0_)/F_m_ (1)

### 2.3. Water Relations Parameters

Water relations parameters were determined after 4, 8 and 12 months from pressure-volume (P-V) curves using two randomly selected seedlings per block and per treatment (32 samples). For each seedling, fresh twigs at the distal end of branches at the same height were selected to avoid possible variations due to differences in hydraulic architecture of forest trees [46]. The development stage and the orientation of the twigs were the same in each treatment. Measurements were conducted using simultaneous pressure chambers (PMS 1000, PMS Instrument Co., Corvallis, OR, USA) and a precision balance to generate the data that were needed to establish pressure–volume curves, according to the method described by Ritchie [54]. Eleven pressure levels (starting at −0.2 MPa down to −5.2 MPa) were applied and each level was maintained for 10 min, as described by Zine El Abidine et al. [46,55]. Before taking the measurements, the twigs of each seedling were placed in distilled water in the dark at 25 °C at 12.00, 14.00, 16.00 and 18.00 h. The length of the saturation period was 20 h. This rehydration until turgor is essential to standardize the relative water content of all samples [46,54]. The measurements were taken over 4 days. Within each day, eight samples were analyzed (four samples per pressure chamber) and 8 P-V curves were generated including two repetitions of each treatment. Each day, samples were chosen randomly among the treatments to avoid rehydration-time effects, as described by Zine El Abidine et al. [46]. The data for the pressure–volume curves were generated at 08.00, 10.00, 12.00 and 14.00 h.

**Figure 1:**
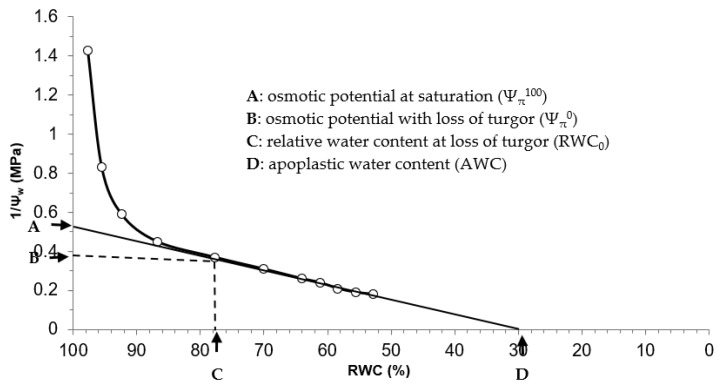
Example of a pressure-volume (P-V) curve generated from different paired measurements [inverse of xylem water potential (1/Ψ_w_) and relative water content (RWC)] indicated by circles. Different variables describing the water relations of the seedlings that were subjected to the four treatments are generated from the P-V curves (indicated by black arrows). These variables include: osmotic potential at saturation (Ψ_π_^100^) osmotic potential with loss of turgor (Ψ_π_^0^), relative water content at loss of turgor (RWC_0_) and apoplastic water content (AWC).

Each pressure–volume curve permitted the estimation of a set of variables describing the water relations of the ectomycorrhizal and non-ectomycorrhizal seedlings, namely, the osmotic potential at saturation (Ψ_π_^100^), the osmotic potential with loss of turgor (Ψ_π_^0^), the relative water content at loss of turgor (RWC_0_), the symplastic water content (SWC), the modulus of elasticity (ε_max_) and the osmotic adjustment (OA). These variables (Ψ_π_^100^, Ψ_π_^0^ and RWC_0_) were determined from the pressure–volume curves (Figure 1), as described by Schulte and Hinckley [45] and Zine El Abidine et al. [46].

Symplastic water content (SWC) was calculated as follows:

SWC (%) = 100 − AWC(2)

Apoplastic water content was determined from the P-V curve. The ε_max_ was calculated as follows [56,57]:

ε_max_ = −Ψ_π_^100^ (1 − AWC)/(1 − RWC_0_) (3)

Osmotic adjustment (OA) was calculated as follows [58]:

OA = Ψ_π_^100^ (control) − Ψ_π_^100^ (treatment) (4)

In the case of the total water potential (Ψ_w_) of small sized plants that were used to generate the pressure–volume curves, only two major components (the osmotic potential (Ψ_π_) and the turgor potential (also termed pressure potential, Ψ_p_) are included, as shown in the following equation:

Ψ_w_ = Ψ_π_ + Ψ_p_ (5)

Ψ_π_ measures the concentration of the solution that is produced by dissolved solutes (always negative). Ψ_p_ represents the hydrostatic pressure that is produced by the inward pressure of cell walls in seedlings or due to water weight (always positive).

### 2.4. Electrolyte Leakage

Electrolyte leakage was measured according to the method described by Blum and Ebercon [59]. Measurements were taken at 4, 8 and 12 months using 5 randomly selected seedlings per block and per treatment (20 seedlings/treatment). Needles were cut into 1 cm long fragments. The fragments (20 fragments per treatment) were washed twice with distilled water, then, were soaked in sterile test tubes containing 15 mL of distilled water in the dark at 40 °C for 1 h. Free conductivity (FC) of the solution was measured after the first incubation using a conductivity meter (Cellox 325, Multiline P3 PH/LF-SET, WTW Gmbh, Weilheim, Germany). The test tubes were incubated in a water bath at 100 °C for 1 h to insure the complete electrolyte leakage. Total conductivity (TC) was measured after the solution reached 25 °C. The rate of electrolytes leakage was calculated as follows [59]:

Electrolyte leakage (%) = (FC/TC) × 100(6)

### 2.5. Statistical Analyses

Analysis of variance (ANOVA) was used to test the significance of the main effect of the treatments being evaluated, the main effect of sampling date and their interactions on the various variables that were measured (gas exchanges, water relations, fluorescence and electrolyte losses, among others) using SPSS 22.0 software (IBM, Armonk, NY, USA). The assumptions of residue normality and variance uniformity for the different variables measured were verified prior to two-way ANOVA [60]. Means comparisons were performed using Tukey’s tests at a 5% significance level. Values are presented as the means ± standard deviation (SD).

## 3. Results

### 3.1. Gas Exchange, Water-Use Efficiency and Chlorophyll Fluorescence

**Figure 2:**
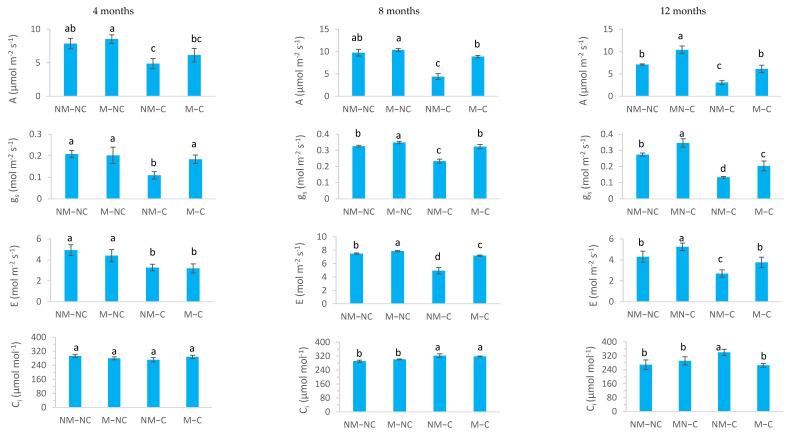
Net photosynthesis (A), stomatal conductance (g_s_), transpiration (E) and intercellular CO_2_ concentration (C_i_) of ectomycorrhizal (M) and non-ectomycorrhizal (NM) *Pinus halepensis* seedlings after 4, 8 and 12 months of growth in contaminated (C) and control or non-contaminated soil (NC). Means (±SD; *n* = 20) with different letters significantly differ from one another based on Tukey’s tests at *p* ≤ 0.05.

There were significant treatment and time effects (*p* < 0.05) on gas exchange variables (A, g_s_, E and C_i_) (Table 1; Figure 2). With the exception of WUE and F_0_, strong and significant interactions between treatment × sampling dates were observed for several physiological variables (Table 1). Under contaminated soil conditions (NM-C and M-C), *P. halepensis* seedlings showed a significant decrease (*p* < 0.05) in A, g_s_ and E. This effect was always more pronounced in non-ectomycorrhizal seedlings (NM-C) during the experiment (Figure 2).

After 12 months of growth, the seedlings were well colonized by the ectomycorrhizal fungus *Rhizopogon* sp. and the extramatrical phase had explored the entire soil volume of the 2 L container. On a microscopic scale, our observations showed that the structures of ectomycorrhizae are characterized by the presence of mantle hyphae and Hartig net hyphae. However, the roots of control seedlings were not colonized by *Rhizopogon* and showed the absence of these microscopic structures. Contaminated soil significantly decreased net photosynthesis in NM-C seedlings after 4 months (38%, *p* = 0.003), 8 months (55%, *p* = 0.0001) and 12 months (57%, *p* = 0.0001), compared to the control (NM-NC). Ectomycorrhizae significantly stimulated net photosynthesis compared to non-ectomycorrhizal seedlings under both treatments (contaminated and non-contaminated soils) (Figure 2). The effect of mycorrhizae on the rate of net photosynthesis was apparent, under longer-term heavy metal stress (>8 months), given that no significant difference was noted between M-C seedlings and the control after 8 months (*p* = 0.153) and 12 months (*p* = 0.758). In addition, M-C seedlings exhibited two-fold higher photosynthesis rates than NM-C after 8 and 12 months (Figure 2).

Non-ectomycorrhizal seedlings (NM-C) had significantly lower stomatal conductance (g_s_) at 4 months (47%, *p* = 0.0001) and 8 months (28%, *p* = 0.0001), compared to the control. After 12 months, g_s_ decreased significantly for both NM-C (52%, *p* = 0.0001) and M-C (26%, *p* = 0.034). This reduction was 1.5 times less pronounced in ectomycorrhizae seedlings (M-C) than in non-mycorrhizal seedlings. In uncontaminated soil, g_s_ was significantly increased by 7% (*p* = 0.042) at 8 months and by 26% (*p* = 0.002) at 12 months for ectomycorrhizal seedlings (M-NC) compared to non-ectomycorrhizal ones (NM-NC) (Figure 2).

Under Pb, Zn and Cd stress, the transpiration rate (E) was significantly reduced for NM-C seedlings at 4 months (33%, *p* = 0.011), 8 months (34%, *p* = 0.0001) and 12 months (37%, *p* = 0.047). This reduction was significantly less accentuated in mycorrhizal seedlings (M-C) throughout the experiment, reaching 35% (*p* = 0.016) at 4 months and 4% (*p* = 0.041) at 8 months, compared to the control. Yet, after 12 months, this effect was not significant (*p* = 0.992). In control soil, the M-NC seedlings showed higher E rates reaching 5% at 8 months (*p* = 0.043) and 22% at 12 months (*p* = 0.005), compared to the control (NM-NC) (Figure 2).

The intercellular CO_2_ concentration (C_i_) of unstressed (NM-NC and M-NC) and stressed (NM-C and M-C) seedlings varied over the experimental period. At 4 months, C_i_ did not exhibit any significant variations (*p* > 0.05) between treatments. After 8 months, C_i_ revealed a significant increase by 11% (*p* = 0.001) for NM-C and by 9% (*p* = 0.014) for M-C. At the end of the experiment, C_i_ increased significantly for NM-C seedlings (26%, *p* = 0.009), while no difference was noted between M-C seedlings and the control (NM-NC) (Figure 2).

**Figure 3:**
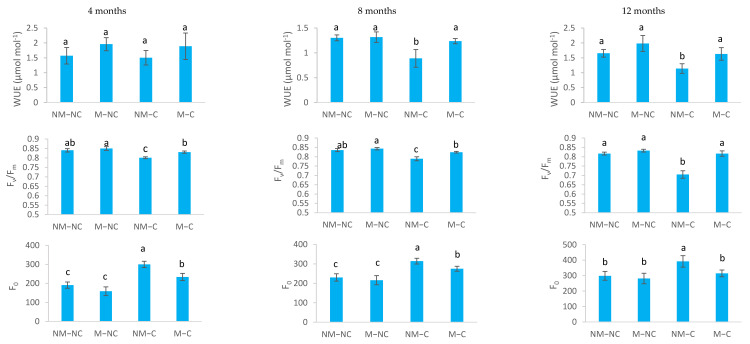
Water-use efficiency (WUE), the yield of photosystem II (F_v_/F_m_) and minimum chlorophyll fluorescence (F_0_) of ectomycorrhizal (M) and non-ectomycorrhizal (NM) *Pinus halepensis* seedlings after 4, 8, and 12 months of growth in contaminated (C) and control soil (NC). Means (± SD; *n* = 20) with different letters significantly differ from one another based on Tukey’s tests at *p* ≤ 0.05.

For water-use efficiency (WUE), only NM-C seedlings showed a significant decrease by 32% at 8 months (*p* = 0.002) and by 31% at 12 months (*p* = 0.018), compared to the control (NM-NC) (Figure 3).

Maximum quantum yield of PSII (F_v_/F_m_) was significantly reduced in non-ectomycorrhizal seedlings (NM-C) under Pb, Zn and Cd stress (Figure 3). F_v_/F_m_ was reduced significantly by 7% (*p* = 0.001) at 4 months, 6% (*p* = 0.014) at 8 months and 14% (*p* = 0.0001) at 12 months, compared to the control (NM-NC). No significant differences in F_v_/F_m_ were noted between mycorrhizal seedlings (M-NC and M-C) and the control (NM-NC) over time (Figure 3). In contrast, exposure to heavy metals (Pb, Zn and Cd) significantly increased minimum chlorophyll fluorescence (F_0_) (Figure 3). NM-C seedlings showed an increase in F_0_, reaching 57% (*p* = 0.0001) at 4 months, 36% (*p* = 0.001) at 8 months and 31% (*p* = 0.036) at 12 months, compared to the control. M-C seedlings showed a less pronounced increase of 22% (*p* = 0.031) and 19% (*p* = 0.048) at 4 and 8 months, respectively, compared to the control. Beyond this period, F_0_ remained constant (*p* = 0.997).

### 3.2. Water Relation Variables

Water relation variables were determined after 4, 8 and 12 months of growth in pots. However, no significant differences (*p* > 0.05) were observed at 4 and 8 months.

**Figure 4:**
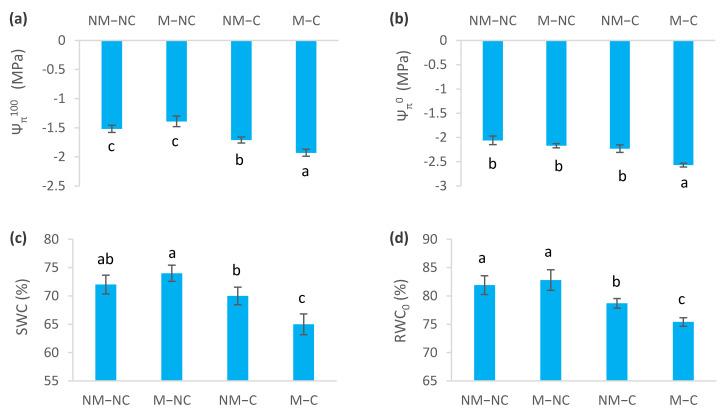

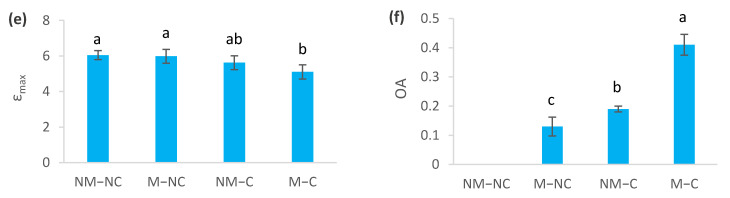
Osmotic potential at saturation (Ψ_π_^100^) (**a**), osmotic potential with loss of turgor (Ψ_π_^0^) (**b**), symplastic water content (SWC) (**c**), relative water content at loss of turgor (RWC_0_) (**d**), modulus of elasticity (ε_max_) (**e**), and osmotic adjustment (OA) (**f**) of ectomycorrhizal (M) and non-ectomycorrhizal (NM) *Pinus halepensis* seedlings after 12 months of growth in contaminated (C) and control soil (NC). Means (± SD; *n* = 8), with different letters indicating significant differences from one another based upon Tukey’s tests at *p* ≤ 0.05.

Prolonged exposure to heavy metals (Pb, Zn and Cd) during 12 months of growth affected water relations variables (*p* < 0.05) (Figure 4). Under heavy metal stress, osmotic potential at full turgor (Ψ_π_^100^) decreased for NM-C by 12% (*p* = 0.030) and for M-C by 27% (*p* = 0.0001), compared to the control (NM-NC) (Figure 4a). Osmotic potential at zero turgor (Ψ_π_^0^) also revealed a decrease by 25% for M-C (*p* = 0.0001), compared to the control (NM-NC). No difference was recorded between NM-C (*p* = 0.054), M-NC (*p* = 0.231) and the control (Figure 4b). Values of Ψ_π_^100^ and Ψ_π_^0^ of mycorrhizal contaminated seedlings (M-C) were significantly more negative than those of seedlings that were grown under the other treatments (NM-NC, M-NC and NM-C). Yet, NM-NC (control) and M-NC seedlings showed higher Ψ_π_^100^ and Ψ_π_^0^ (less negative) values (Figure 4a,b).

Symplastic water content (SWC) decreased for mycorrhizal seedlings (M-C) by 10% (*p* = 0.001), compared to the control (NM-NC). No significant difference was observed between M-NC (*p* = 0.696), NM-C (*p* = 0.262) and the control (NM-NC) (Figure 4c). RWC_0_ was significantly reduced in NM-C by 4% (*p* = 0.031) and M-C by 8% (*p* = 0.001), compared to the control (Figure 4d). The modulus of elasticity (ε_max_) decreased by 16% for ectomycorrhizal seedlings M-C (*p* = 0.005), but it did not significantly differ from NM-C (Figure 4e). Mycorrhizal seedlings (M-C) showed 2.15-fold higher osmotic adjustment (OA) than non-mycorrhizal ones (NM-C), and 3.15-fold higher than M-NC (Figure 4f).

### 3.3. Electrolyte Leakage

**Figure 5:**
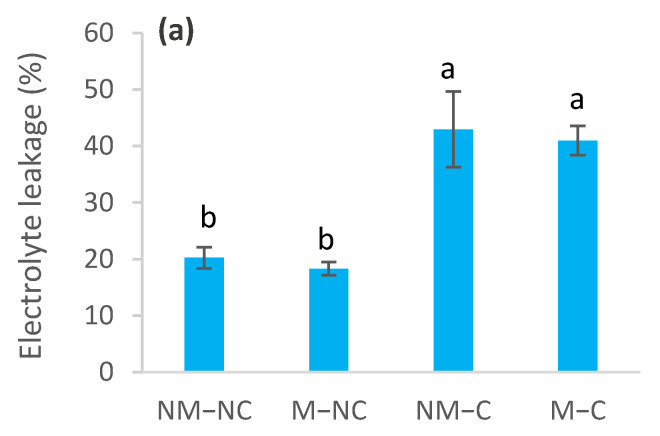

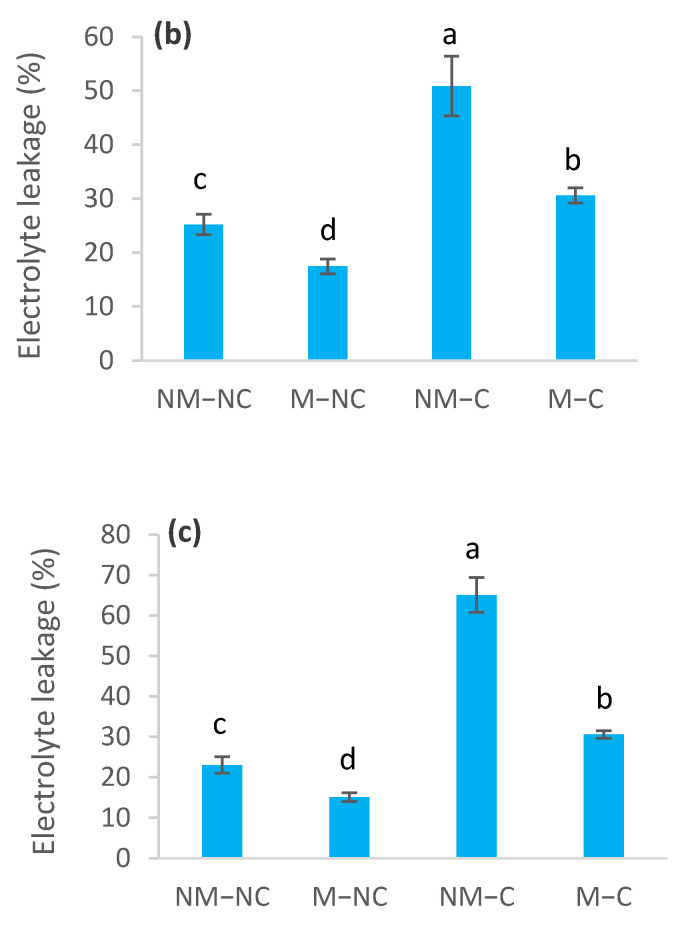
Electrolyte leakage of ectomycorrhizal (M) and non-ectomycorrhizal (NM) *Pinus halepensis* seedlings after 4 (**a**), 8 (**b**) and 12 months (**c**) of growth in contaminated (C) and control soil (NC). Means (± SD; *n* = 4) with different letters significantly differ from one another based on Tukey’s tests at *p* ≤ 0.05.

The effects of heavy metals on membrane permeability varied throughout the experiment, depending upon the presence or absence of ectomycorrhizal fungi (Figure 5). The sampling date had no effect (*p* = 0.141) on electrolyte leakage (Table 1). In contaminated soil, electrolyte leakage was increased for both ectomycorrhizal (M-C) and non-ectomycorrhizal (NM-C) seedlings, compared to the control (NM-NC). The increase in electrolyte leakage for NM-C reached 112% (*p* = 0.0001) at 4 months, 102% (*p* = 0.0001) at 8 months and 183% (*p* = 0.0001) at 12 months, compared to the control (Figure 5). No difference (*p* = 0.753) was noted between NM-C and M-C at 4 months (Figure 5a). Compared to the control (NM-NC), ectomycorrhizal seedlings (M-C) had significantly lower electrolyte leakage rates, reaching 102% (*p* = 0.0001) at 4 months, 21% (*p* = 0.031) at 8 months and 33% (*p* = 0.001) at 12 months (Figure 5). In control soil (NC), leakage of electrolytes was reduced for M-NC after 8 months (31%, *p* = 0.003) and 12 months (34%, *p* = 0.0001), compared to the control. M-NC seedlings generally exhibited the lowest values of electrolyte leakage throughout the experiment (Figure 5).

## 4. Discussion

The use of ectomycorrhizal *P. halepensis* seedlings that were infected with *Rhizopogon* sp. in the presence of soil contaminated with heavy metals significantly improved gas exchange and water relations (Figure 2, Figure 3 and Figure 4). After 12 months of growth, the mycorrhizae also conferred on Aleppo pine seedlings an osmotic adjustment and a high elasticity allowing the plants to survive, grow and maintain various physiological processes (Figure 2, Figure 3, Figure 4 and Figure 5) despite the presence of extremely high concentrations of heavy metals in the soil (Pb, Zn and Cd). Our previous results [47] showed that after 12 months of growth in contaminated soil (NM-C) with heavy metals (Pb, Zn, and Cd), shoot and root dry masses of *P. halepensis* seedlings were reduced compared to the control (NM-NC), while no differences were observed for M-C compared to the control (NM-NC). Prolonged exposure to heavy metals primarily affects seedling growth and significantly decreases nutrient uptake, water-use efficiency, photosynthetic activity and cell membrane integrity [19,22,25,29]. In contrast, other studies showed that mycorrhizal fungi alleviate metal toxicity by improving physiological mechanisms and adaptation of host plants [23,61]. This observation is consistent with the current research indicating a significant increase in photosynthetic rate among ectomycorrhizal seedlings (M-C) to a level similar to seedlings under controlled conditions (NM-NC) (Figure 2). The increase in M-C was two times greater than non-ectomycorrhizal seedlings (NM-C) after 8 and 12 months of exposure to heavy metal contaminated soil (Figure 2). Other results have demonstrated an adverse effect of heavy metals on gas exchange parameters [25,62,63]. This was evaluated through analysis of maximum quantum yield of PSII photochemistry (F_v_/F_m_), which is considered to be a critical parameter of plant photosynthetic performance and a useful tool for evaluating plant tolerance to heavy metal toxicity. Our results revealed a significant F_v_/F_m_ reduction under Pb, Zn and Cd toxicity, particularly in non-ectomycorrhizal seedlings (NM-C), while M-C seedlings always exhibited higher values (Figure 3). For non-ectomycorrhizal seedlings, the decline in F_v_/F_m_ ratio suggests that photodamage and photoinhibition occurred to PSII [64,65]. This effect significantly decreases net photosynthesis as was observed in non-ectomycorrhizal seedlings (Figure 2). Under controlled conditions, net photosynthesis was positively correlated with internal CO_2_ concentration [66]. Yet, an increase in internal CO_2_ (C_i_) concentrations may be associated with low net uptake of CO_2_. Therefore, photosynthetic limitation was posed by internal conductance to CO_2_ movement in seedlings that were exposed to contaminated soil (NM-C and M-C) after 8 months (Figure 2). It has been shown that Cd stress is able to increase CO_2_ concentrations in the intercellular spaces of the mesophyll [67]. High internal CO_2_ levels can be explained by the decreased capacity of chloroplasts to assimilate CO_2_ [68]. In contrast, ectomycorrhizae enabled the seedlings (M-C) to withstand metal toxicity and to maintain C_i_ concentrations that were similar to the control after 12 months of growth (Figure 2). Ectomycorrhizae also increased transpiration (E) rates in seedlings (M-C) after 8 and 12 months of exposure to excess of Pb, Zn and Cd (Figure 2). Similarly, Han et al. [42] demonstrated an increase in photosynthetic and transpiration rates in ectomycorrhizal hybrid poplar (*Populus alba* × *tremula* var. *glandulosa*) seedlings that had been subjected to high concentrations of Cd.

Mycorrhizal plants often exhibit higher stomatal conductance (g_s_) than non-mycorrhizal ones [69]. Our results showed that stomatal conductance of ectomycorrhizal seedlings (M-C) is not different to the control (NM-NC), but it is always significantly greater than the non-ectomycorrhizal seedlings (NM-C) throughout the growing period (12 months) (Figure 2). Heavy metals negatively affect absorption of mineral nutrients due, in part, to stomatal closure, which leads to the decrease in CO_2_ uptake and photosynthesis [33,70,71,72]. Other major positive effects of ectomycorrhizae on water relations and gas exchange parameters are associated with (i) increasing soil root exploration by extending the extraradical phase (hyphae and mycelial strands) of ectomycorrhizal fungi that are capable of penetrating soil micropores, which are not accessible to the roots, to extract water [37,39,40,73,74,75,76]. (ii) The formation of mycelial strands or rhizomorphs by certain ectomycorrhizal fungi, as is the case in our study (*Rhizopogon* sp.), which are capable of transporting large quantities of water [37,74], and the extraradical mycelium of ectomycorrhizal fungi, by itself (without the roots), make it possible to satisfy the moisture requirements of the seedling in order to maintain photosynthesis and transpiration [14,38]. (iii) Furthermore, water stress is reduced at the soil-root interface [39], together with (iv) improvement of soil structure and soil water reserves depending upon the density and expanse of the fungal hyphae in the soil [75,77,78]. Our previous results have shown that ectomycorrhizae significantly increase the dry root masses of *P. halepensis* seedlings [47]. The development of root systems in ectomycorrhizal plants was linked to higher rates of CO_2_ uptake and greater WUE in stone pine (*Pinus pinea* L.) [79]. It should be noted that ectomycorrhizal seedlings (M-C) had the most rapid and efficient recovery from the harmful effects of heavy metals compared to the non-ectomycorrhizal ones (NM-C) (Figure 2 and Figure 3). The recovery time was associated to the rapid growth of plants, which causes a reduction in internal metal concentrations due to a dilution effect [28]. Our previous results showed that ectomycorrhizal *P. halepensis* seedlings exhibit higher growth and lower Pb, Zn and Cd uptake than non-ectomycorrhizal seedlings [47], which demonstrates the substantial benefits of ectomycorrhizal fungi to their hosts under multi-metal stress.

Stomatal closure disrupts the flow of sap and, consequently, the water relations of the seedlings [80]. Therefore, the importance is demonstrated for osmotic adjustment (OA) as an adaptation mechanism [81]. This is consistent with our findings showing a substantial increase in OA for ectomycorrhizal (M-C) seedlings that was two-fold higher than non-ectomycorrhizal ones (NM-C) at the end of the experiment (Figure 4f). The increase in osmotic adjustment may be associated with decreases in osmotic potentials (Ψ_π_^100^ and Ψ_π_^0^) (Figure 4a,b), suggesting that mycorrhizae can give plants increased resistance to various heavy metals. A greater decline in Ψ_π_^100^ and Ψ_π_^0^ for ectomycorrhizal seedlings (M-C), together with greater osmotic adjustment (Figure 4), may grant them the ability to absorb water from contaminated soil, even when the former is hardly available due to effects of contamination [18]. As a result, M-C seedlings can maintain their turgor for a long time before reaching the loss of turgor point [46], which is consistent with lower values of RWC_0_ and increased elasticity of cell membranes (Figure 4). This allows gas exchange (stomata conductance and net photosynthesis) to be maintained for longer periods [52], as was observed in this study. The positive effects of these significant improvements in physiological processes were reflected on several growth parameters [47].

In contrast, Muhsin and Zwiazek [82] revealed that ectomycorrhizae increase apoplastic water transport and root hydraulic conductivity in American elm (*Ulmus americana* L.) seedlings, and suggested that this is related to decreased resistance to the flow of water from the apoplast through ectomycorrhizal hyphae. The increased root water flow in mycorrhizal seedlings may be related to the nutritional and metabolic effects of mycorrhizae on the activity of root water channels [82]. It has been demonstrated that ectomycorrhizal fungi improve water transport and water relations of seedlings [37,38,39] through the improvement in the mineral nutrition of plants. Phosphorus and potassium, especially, are two key elements that are involved in active adjustments [73]. Several ectomycorrhizal fungi improve the osmotic adjustment of their host seedling’s cells. This improvement came from the synthesis of organic acids and accumulation of mineral nutrients [83,84]. For example, high concentrations and contents of nitrogen, phosphorus and calcium increase the rate of net photosynthesis [48,76,85], which could increase water-use efficiency compared to non-ectomycorrhizal ones (NM-C), as was noted in the present study (Figure 3). Indeed, our recent findings revealed that ectomycorrhizal association of *P. halepensis* seedlings improved mineral nutrition, particularly in terms of nitrogen and calcium [47]. This last mineral nutrient (Ca) stimulates photosynthesis, cell division, cell wall rigidity and absorption of major nutrients (N, P and K) [86,87,88]. Furthermore, osmotic adjustments are known to prevent oxidative damage [26,27,89]. This effect becomes more important in the presence of mycorrhizae [90]. This may explain the low electrolyte leakage rates that were sustained by ectomycorrhizal contaminated (M-C) seedlings (Figure 5b,c). Reduction in electrolyte leakage may be associated with the cell conservation of water absorption and transport structures within the seedlings [27]. Membrane stability of ectomycorrhizal seedlings may be further linked to the decrease in the modulus of elasticity (ε_max_) (Figure 4e), which provides more flexibility and stability to the membranes [46]. This flexibility provides the plant with the possibility of undergoing significant variation in the water content of the apoplast without affecting the dynamic structure of the cell walls [91]. This is confirmed by the significant decrease in the symplastic water content (SWC) in ectomycorrhizal (M-C) seedlings, compared to the control (NM-NC) and the non-ectomycorrhizal ones (NM-C) (Figure 4c). As such, the influence of metal contamination on membrane stability was not associated with heavy metal exposure time, unlike the other variables (Table 1). This response is probably due to the protection that is offered by the ectomycorrhizal symbiosis, which confers greater resistance to stress, thereby lowering the occurrence of stress symptoms, such as membrane stability maintenance and decreased osmo-protectant production [92].

## 5. Conclusion and Research Needs

The present study emphasizes the importance of ectomycorrhizae in enhancing physiological processes in *P. halepensis* seedlings that are subjected to heavy metal contaminated soil, and supports the use of a mycorrhizoremediation approach in reforestation and rehabilitation of heavy metal contaminated sites. Our results, combined with our previous results [47], suggest that the use of ectomycorrhizal fungi is among the promising practices that would improve the morphophysiological quality of tree seedlings produced in forest nurseries, their performance and their tolerance to multiple heavy metal stresses (Pb, Zn and Cd). Therefore, further study is warranted regarding the combined effects of heavy metals and ectomycorrhizae on the mineral status of the seedlings using vector analysis of foliar nutrients and biomass.

## Figures and Tables

**Table 1:** Significance levels of treatment effects (T), date effects (D) and their interaction (T × D).

	Significance		
Variable	Treatment (T)	Date (D)	T × D
A (µmol m^−2^ s^−1^)	0.0001	0.0001	0.007
g_s_ (mol m^−2^ s^−1^)	0.0001	0.0001	0.0001
E (mol m^−2^ s^−1^)	0.0001	0.0001	0.001
C_i_ (µmol mol^−1^)	0.0001	0.001	0.0001
WUE (µmol mol^−1^)	0.0001	0.0001	0.179 *
F_v_/F_m_	0.0001	0.0001	0.0001
F_0_	0.0001	0.0001	0.608 *
Electrolyte leakage (%)	0.0001	0.141 *	0.0001
Ψ_π_^100^ (MPa)	0.0001	-	-
Ψ_π_^0^ (MPa)	0.0001	-	-
SWC (%)	0.002	-	-
RWC_0_ (%)	0.002	-	-
ε_max_	0.327 *	-	-
OA	0.0001	-	-

The experimentation included four treatments [NM-NC (non-mycorrhizal seedlings + non-contaminated soil (control soil)), M-NC (mycorrhizal seedlings + non-contaminated soil), NM-C (non-mycorrhizal seedlings + contaminated soil) and M-C (mycorrhizal seedlings + contaminated soil)] and three sampling dates (4 months, 8 months and 12 months). A: net photosynthesis; g_s_: stomatal conductance; E: transpiration; C_i_: intercellular CO_2_ concentration; WUE: water-use efficiency; F_0_: minimum chlorophyll fluorescence; F_v_/F_m_: the potential quantum yield of photosystem II; Ψ_π_^100^: osmotic potential at saturation; Ψ_π_^0^: osmotic potential with loss of turgor; RWC_0_: relative water content at loss of turgor; SWC: symplastic water content; ε_max_: modulus of elasticity; OA: osmotic adjustment. *: non significant.

## Data Availability

Not applicable.
